# Digitally supported program for type 2 diabetes risk identification and risk reduction in real-world setting: protocol for the StopDia model and randomized controlled trial

**DOI:** 10.1186/s12889-019-6574-y

**Published:** 2019-03-01

**Authors:** Jussi Pihlajamäki, Reija Männikkö, Tanja Tilles-Tirkkonen, Leila Karhunen, Marjukka Kolehmainen, Ursula Schwab, Niina Lintu, Jussi Paananen, Riia Järvenpää, Marja Harjumaa, Janne Martikainen, Johanna Kohl, Kaisa Poutanen, Miikka Ermes, Pilvikki Absetz, Jaana Lindström, Timo A. Lakka, on behalf of the StopDia study group, Tiina Laatikainen, Kennet Harald, Markku Peltonen, Pekka Jousilahti, Katri Hemiö, Maliheh Nekouei Marvi Langari, Eeva Virtanen, Saara Pentikäinen, Johanna Leväsluoto, Adil Umer, Juha Leppänen, Samuli Heinonen, Elina M. Mattila, Kari Jalkanen

**Affiliations:** 1 Department of Clinical Nutrition, Institute of Public Health and Clinical Nutrition, University of Eastern Finland, 70210 Kuopio, Finland; 2 Clinical Nutrition and Obesity Center, Kuopio University Hospital, Kuopio, Finland; 3 Department of Medicine, Endocrinology and Clinical Nutrition, Kuopio University Hospital, Kuopio, Finland; 4 Institute of Biomedicine, School of Medicine, University of Eastern Finland, Kuopio, Finland; 5 Public Health Solutions, National Institute for Health and Welfare, Helsinki, Finland; 6 VTT Technical Research Centre of Finland Ltd., Espoo, Finland; 7 School of Pharmacy, University of Eastern Finland, Kuopio, Finland; 8 Collaborative Care Systems Finland, Helsinki, Finland; 9 Department of Clinical Physiology and Nuclear Medicine, Kuopio University Hospital, Kuopio, Finland; 10 Kuopio Research Institute of Exercise Medicine, Kuopio, Finland

**Keywords:** Type 2 diabetes, Lifestyle intervention, Prevention, Randomized controlled trial, Digital health behavior change intervention, Scalability, Effectiveness

## Abstract

**Background:**

The StopDia study is based on the convincing scientific evidence that type 2 diabetes (T2D) and its comorbidities can be prevented by a healthy lifestyle. The need for additional research is based on the fact that the attempts to translate scientific evidence into actions in the real-world health care have not led to permanent and cost-effective models to prevent T2D.

The specific aims of the StopDia study following the Reach, Effectiveness, Adoption, Implementation, and Maintenance (RE-AIM) framework are to 1) improve the Reach of individuals at increased risk, 2) evaluate the Effectiveness and cost-effectiveness of the digital lifestyle intervention and the digital and face-to-face group lifestyle intervention in comparison to routine care in a randomized controlled trial (RCT), and 3) evaluate the Adoption and Implementation of the StopDia model by the participants and the health care organizations at society level. Finally, we will address the Maintenance of the lifestyle changes at participant level and that of the program at organisatory level after the RCT.

**Methods:**

The StopDia study is carried out in the primary health care system as part of the routine actions of three provinces in Finland, including Northern Savo, Southern Carelia, and Päijät-Häme. We estimate that one fifth of adults aged 18–70 years living in these areas are at increased risk of T2D. We recruit the participants using the StopDia Digital Screening Tool, including questions from the Finnish Diabetes Risk Score (FINDRISC). About 3000 individuals at increased risk of T2D (FINDRISC ≥12 or a history of gestational diabetes, impaired fasting glucose, or impaired glucose tolerance) participate in the one-year randomized controlled trial. We monitor lifestyle factors using the StopDia Digital Questionnaire and metabolism using laboratory tests performed as part of routine actions in the health care system.

**Discussion:**

Sustainable and scalable models are needed to reach and identify individuals at increased risk of T2D and to deliver personalized and effective lifestyle interventions. With the StopDia study we aim to answer these challenges in a scientific project that is fully digitally integrated into the routine health care.

**Trial registration:**

ClinicalTials.gov. Identifier: NCT03156478. Date of registration 17.5.2017.

## Background

Previous clinical trials have shown the efficacy of lifestyle interventions in the prevention of type 2 diabetes in different populations [1–4] and the sustainability of the beneficial effects for several years after the discontinuation of the intervention [5, 6]. The efficacy of such interventions depends strongly on the lifestyle changes adopted, which emphasises successful health behavior change in the prevention of type 2 diabetes [5]. Importantly, sustainable and scalable models are needed to reach and identify individuals at increased risk of type 2 diabetes and to deliver personalized and effective lifestyle interventions.

The implementation of evidence-based approaches for reaching and identifying individuals at increased risk and preventing type 2 diabetes as permanent practices in the health care system and the society is still a challenge. Individuals at increased risk can be identified based on a history of non-diabetic dysglycaemia or gestational diabetes [7, 8] but also using validated risk screening tools, such as the Finnish Diabetes Risk Score (FINDRISC) [9]. However, such risk screening tools are not yet utilized systematically in the identification of individuals at increased risk of type 2 diabetes in the health care system and the society.

Many seminal lifestyle intervention studies, such as the Diabetes Prevention Study in Finland [1] and the Diabetes Prevention Program in the United States [2] as well as most of the implementation studies following these efficacy trials [10, 11] have been based on face-to-face counselling either individually or in groups. However, individual counseling is resource intensive and may not be feasible for most health care systems [10]. Group counseling demands less resources but requires special skills [12] which may limit its use in the health care system. Although digital interventions are more scalable than face-to-face interventions, adherence to digital interventions remains a challenge [13, 14].

The Stop Diabetes (StopDia) study was set to create and implement evidence-based and digitally supported strategies for the prevention of type 2 diabetes at population level in three provinces in Finland. This paper describes the protocol for a digital identification and recruitment of individuals at increased risk and a randomized controlled trial on the effectiveness of a digital and face-to-face group lifestyle intervention in reducing the risk of type 2 diabetes compared with usual care. Importantly, the risk identification and the StopDia lifestyle interventions are fully integrated in the Finnish health care system to facilitate their long-term implementation.

## Aims of the study

We will conduct a comprehensive process and outcome evaluation of the StopDia study by following the **Reach**, **Effectiveness**, **Adoption**, **Implementation**, and **Maintenance** (RE-AIM) framework designed for assessing the public health impact of health promotion policy or programmes [15].

The specific aims of the StopDia study according to the RE-AIM framework are to evaluate:

1. **Reach** of the StopDia model in terms of coverage of risk screening and uptake of the intervention by the target population. We will evaluate the efficiency of different approaches in identifying individuals at increased risk of type 2 diabetes and study how the identification of individuals at increased risk translates into participation in the interventions in different population groups.
2. **Effectiveness and cost-effectiveness** of the digital lifestyle intervention and the digital and face-to-face group lifestyle intervention in comparison to routine care on the outcomes in a randomized controlled trial of the StopDia study.
3. **Adoption and Implementation** of the StopDia model by measuring adherence to the digital and face-to-face group lifestyle intervention (participant level) and activities related to the prevention of type 2 diabetes in the three provinces and by the health care organizations (societal and setting level) during the randomized controlled trial.
4. **Maintenance** of the lifestyle changes (participant level) and the continuation of the program activities (societal and setting level) after the randomized controlled trial (the process not described in detail in this report).

## Methods

### Study population and design

The StopDia study is carried out in the primary health care system as part of the routine actions of three provinces in Finland, including Northern Savo, Southern Carelia, and Päijät-Häme. The population in these areas encompass 580,000 adults aged 18–70 years. We estimate that 116,000 (one fifth) of these adults are at increased risk of type 2 diabetes (FINDRISC ≥12 or history of gestational diabetes, impaired glucose tolerance, or impaired fasting glucose) based on the results of a national population-based survey [16]. We will first describe below the protocol for screening, reaching, and identifying individuals at increased risk of type 2 diabetes, and thereafter the protocol for the randomized controlled trial.

### Protocol for evaluating reach: Recruitment and screening of individuals at increased risk

#### Recruitment and screening

The participants are recruited using the StopDia Digital Screening Tool that was developed for the StopDia study and is in Finnish. The screening tool includes questions from the FINDRISC (age, body weight, body height, waist circumference, daily physical activity, the daily consumption of vegetables, fruits, and berries, a history of regular use of blood pressure medication, a history of elevated blood glucose, a family history of diabetes) [9]. The screening tool also includes questions on gender, education, the exclusion criteria (prevalent type 1 or type 2 diabetes, current pregnancy and breastfeeding, an active cancer or less than six months from cancer treatment), and the inclusion criteria (a postal code to confirm living in one of the three provinces, a possibility to use a computer, smartphone, or tablet with an internet connection, having a phone number of own, having adequate Finnish language skills). Moreover, the individuals are asked where they learned about the StopDia study and who encouraged them to fill out the StopDia Digital Screening Tool.

We test the effectiveness of various communication channels, including social media, newspapers, radio, TV, websites, health care and social service units, and community pharmacies in increasing the awareness about the StopDia study and in engaging people to visit the StopDia Digital Screening Tool. We collaborate with municipal services, employers, patients associations, and other non-governmental organizations to increase the coverage and uptake of screening. Moreover, we use digital (emails, SMS messages, videos, banners), printed (posters, flyers), and other material (the FINDRISC waist circumference measuring tape) created by the StopDia group to support recruitment digitally as well as in public events and supermarkets. Moreover, we perform recruitment in food banks to reach individuals from lower socioeconomic groups since they are known to be a vulnerable population that is usually difficult to reach in health-related studies [17, 18].

#### Data collection at the recruitment and screening phase

The answers in the StopDia Digital Screening Tool as well as the date and time of the initiation of responding to the questions are stored anonymously in a digital database, because the individuals have not signed the informed consent to participate in the study at this phase. We also keep a log on the media activity and other recruitment activities of the StopDia study and collect data on daily page views at the digital screening website using the Google Analytics service to explore the effectiveness of different recruitment methods and activities. The demographics and type 2 diabetes risk factors of the respondents are compared with those of the general population to investigate the generalizability of our findings.

### Protocol for evaluating effectiveness and cost-effectiveness: The randomized controlled trial

#### Participants

**Fig. 1:**
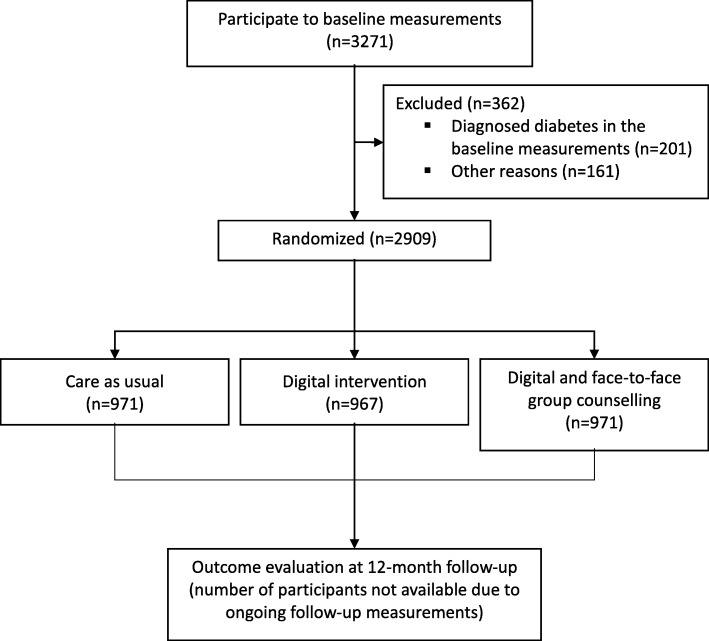
Flow diagram of the Stop Diabetes intervention study

We recruit about 3000 individuals to participate in the one-year randomized controlled trial (ClinicalTrials.com, NCT03156478). Detailed number of the randomized study participants and study design of the ongoing randomized controlled trial are described in the Fig. 1. The inclusion criteria are 1) age 18–70 years; 2) increased risk of type 2 diabetes based on the FINDRISC score ≥ 12 points or a history of gestational diabetes or repeated impaired fasting glucose (fasting plasma glucose 6,1–6,9 mmol/l) or impaired glucose tolerance (2-h plasma glucose 7,8–11,0 mmol/l in 2-h oral glucose tolerance test [OGTT]); 3) living in the province of Northern Savo, or Southern Carelia, or Päijät-Häme; 4) possibility to use a computer, smartphone, or tablet with internet connection; 5) having a phone number of their own; and 6) having adequate Finnish language skills. The exclusion criteria are 1) type 1 or type 2 diabetes; 2) pregnancy or breastfeeding; and 3) active cancer or less than six months from cancer treatment. After the one-year randomized controlled trial, we aim to follow the participants for a total of 20 years.

#### Examination visits and randomization

Individuals identified to be eligible by the StopDia Digital Screening Tool are automatically invited to participate in the randomized controlled trial. On the StopDia webpages, an individual who is willing to participate is given instructions on how to contact a nurse in a local health care center for examination visits.

At the first examination visit, the participant signs an informed consent, and the nurse measures body weight, body height, waist circumference, and blood pressure using calibrated devices, makes a referral to the laboratory measurements, and gives a blood sampling kit and tubes to the participant for the laboratory measurements. The sampling kit and the tubes are identified with numerical identification codes. After the first examination visit, the participant receives a personal link by email to a web-based StopDia Digital Questionnaire developed using the LimeSurvey platform (LimeSurvey GmbH, Hamburg, Germany). The participant is instructed to complete the questionnaire within two weeks.

The second examination visit is carried out in the laboratory of the local health care center. The participant is instructed to fast for 12 h, avoid strenuous physical activity, abstain from drinking alcohol for 24 h, and avoid smoking in the morning before the visit. Trained laboratory nurses take fasting blood samples for routine laboratory tests, explained in detail in Assessments, and blood samples during the 2-h OGTTs. These blood samples are stored and analysed at local clinical laboratories. Additional blood samples for research purposes, taken with a sampling kit, are frozen and transported to the laboratory of the StopDia research group at the University of Eastern Finland.

The participants who meet the inclusion criteria and have no exclusion criteria according to the StopDia Digital Screening Tool, have filled out the StopDia Digital Questionnaire, have given blood samples, and have no type 2 diabetes according to the results of the 2-h OGTT are randomly assigned into one of the three study groups (digital lifestyle intervention, digital and face-to-face group lifestyle intervention, control) with 1:1:1 allocation using a computerized randomization system. Individuals whose 2-h OGTT indicates the diagnosis of type 2 diabetes are directed to diabetes care following the normal principles of the provinces.

After randomization, the participants receive an electronic information letter, including instructions how to proceed in the study according to their study group and a detailed description of the contents of the interventions, by an e-mail and a SMS message. Individuals in the control group receive electronic information about lifestyle risk factors of type 2 diabetes and recommendations on a healthy diet and physical activity in accordance with international and national recommendations for nutrition [19, 20] and physical activity [21–23].

At the end of the one-year randomized controlled trial, the participants are sent an e-mail and a SMS message to remind them to make an appointment for the examination visit carried out by nurses at local health care centers. The participants are also given a personal link to the follow-up StopDia Digital Questionnaire and are referred to the laboratory visit, similarly to the baseline.

#### Lifestyle interventions

##### Theoretical background of interventions

The lifestyle interventions delivered through the digital application and the face-to-face group coaching share the same overarching behavior change theory, the Self-Determination Theory [24]. This approach emphasizes the role of autonomous motivation, perceived competence or self-efficacy, and social relatedness in lifestyle changes. In the face-to-face group coaching, the lifestyle changes are mainly targeted through deliberative processes with the Behavior Change Techniques [25] from the self-regulation theories [26], such as self-monitoring, goal setting, as well as action and coping planning. The aspects of the model of eating competence [27], such as eating attitudes, food acceptance, internal regulation of food intake, and management of eating context, are also taken into account. The digital intervention utilizes a habit-based approach to behavior change [28, 29] by providing the participans a broad selection of context-specific minimum-effort behaviors, called BitHabit, to browse and choose from. The participants are reminded and prompted to self-monitor their performance based on the selected BitHabit and are rewarded through the visible accumulation of successful performance. Social relatedness is targeted by informing the participants about other users’ behavior in their BitHabit selection and performance. While the goal of the face-to-face group coaching is to increase motivation and self-efficacy in behavior change, the goal of the digital intervention is to facilitate the automatization of the BitHabit into daily practices that do not require much motivation or effort.

##### Lifestyle goals of interventions

The digital and face-to-face group intervention rely on the scientific evidence on the role of lifestyle factors in the prevention of type 2 diabetes [1–6]. Thus, the main lifestyle goals of the intervention are to improve diet, increase physical activity, decrease sedentary behavior, reduce excess body weight, improve sleep, cessate smoking, and moderate alcohol consumption (Table 1). The goals for diet are based on the Nordic and Finnish Nutrition Recommendations [19, 20], physical activity and sedentary behavior on the international physical activity guidelines [21–23], sleep on the consensus statement for sleep to support health [30], and smoking and alcohol consumption on the evidence for their adverse effects on the risk of type 2 diabetes [31, 32]. The lifestyle intervention also aims at improving mental wellbeing, reducing psychological distress, and promoting positive mood and stress management as they have been related to diabetes and may also impact the ability to maintain a healthy lifestyle [33].

**Table 1:** The main lifestyle goals of the intervention

Lifestyle target	Lifestyle recommendation
Diet	
Dietary score	Improvement in diet score
Breakfast	Have a breakfast on 5 week days
Roots and non-root vegetables	≥ 3 portions^a^ of roots and non-root vegetables per day
Fruits and berries	≥ 2 portions^b^ of fruits and berries per day
Fish	Fish ≥2 times per week
Red and processed meat	Red and processed meat ≤5 times per week
Milk products	4 portions^c^ of low-fat and fat-free milk, sour milk and yoghurt (≤1% fat), low-fat cheese (≤17% fat) and milk products with no or low in sugar per day
Vegetable oils and vegetable oil based spreads	Vegetable oils and vegetable oil based spreads on bread, cooking, and in salad dressings
Grain products	≥6 portions^d^ of grain products (> 50% whole grain) per day in women and ≥ 9 portions^d^ per day in men
Nuts, seeds, and almonds	About 30 g of natural nuts, seeds, and almonds per day
Sugar containing beverages	≤2 dl of sugar containing beverages per day
Alcohol beverages	≤1 portion^e^ of alcohol beverages per day in women and ≤ 2 portions^e^ per day in men
Physical activity	
Physical activity	≥30 min of at least moderate intensity physical activity per day on at least five days a week
Sedentary behavior	Decreased sedentary behavior
Other	
Sleeping time	≥7 h of sleeping per day
Smoking	To quit or at least decrease smoking
Body weight	Weight loss
Mood	Increased attention to positive mood inducing factors
Stress	Increased use of relaxation techniques

^a^1 portion = 1 dl grated vegetables, salad or cooked vegetables, 1 average size carrot or 2 tomatoes

^b^1 portion = 1 dl berries or 1 average size fruit

^c^1 portion = 2 dl liquid dairy products (e.g. milk, sour milk, yoghurt) or 2 slices cheese

^d^1 portion = 1 slice of bread, ½ roll, 1,5 dl porridge or ½ dl muesli

^e^1 portion = 12 cl wine, 33 cl medium strength beer or 4 cl spirits

##### Digital intervention

The habit-based digital lifestyle intervention, the BitHabit, is implemented as a web application. The concept of the habit-based lifestyle intervention was developed as a collaborative process by researchers representing digital health, psychology, nutrition, exercise medicine, and medicine, together with the lay representatives of the target group. The front-end software is independent of operating environments and can be deployed on any web-server and operating system. The back-end software requires the Java Runtime Environment. During the study, both application services will be deployed on the Microsoft Azure Cloud computing platform (Microsoft Corporation, Redmond, USA). The application can be used with all smart devices, such as computers, tablets, and smart phones, and does not require installing a separate application. After randomization to the respective study groups the participants receive a personalized link by email and SMS message and can access the application directly by clicking the link. During the first 10 min of use, the application gives the participant instructions through pop-up messages. The participants can also use a user manual any time by clicking a questionmark icon on the screen. The participants are instructed to use the application throughout the one-year intervention period.

The main functionalities of the BitHabit application are 1) browsing behavioral suggestions and selecting those that the users want to perform, 2) daily self-monitoring of the selected behaviors, and 3) getting summary feedback for habit formation in each of the 13 lifestyle categories (Table 2). The application also provides information on other users’ selections in an anonymous format through pop-up messages. Reminders are sent by emails and SMS messages if the user does not select any habits, add any performance, start using the application within two days after the first uptake message, or use the application for seven days. The application also has an additional self-learning section that provides reliable information on the prevention of type 2 diabetes.

**Table 2:** The functionalities and contents of the web application

Functionality	Content
Browsing habits	Users can browse and select habits in 13 lifestyle categories.
Inspecting habits	Users can inspect the detailed description of habits, including a brief title, a short description, and a health fact.
Selecting habits	Users can select habits to be repeated on a daily basis.
Reporting performances	Users can report habit performances on a daily basis.
Reflecting on own activities	Users can view a summary, including the number of selected habits and performances per 13 categories.
Getting information on other users’ activities	Users can see a summary of other users’ habits and performances in an anonymous format in pop-up messages.
Getting information on the prevention of type 2 diabetes	Users can read information on the prevention of type 2 diabetes.
Getting reminders	Users receive reminders by emails and SMS messages if they do not select any habits, add any performance, start using the application within two days after the first uptake message, or use the application for seven days.

The BitHabit application provides an extensive evidence-based habit library that was developed by translating lifestyle guidelines and recommendations into simple habit-forming suggestions of behaviors that can easily be adopted into daily life. Each habit has a brief title, a more detailed description, and a health fact derived from the existing knowledge. The library consists of 489 behavioral suggestions divided into 13 lifestyle categories, including meal frequency, vegetables, dietary fat, grain products, sugar, alcohol and other drinks, conditioning physical activity, everyday physical activity, sedentary behavior, sleep, stress management, positive mood, and non-smoking. The order of these categories in the user interface is determined by a few questions on health behaviors that are asked at the first login. Categories where improvement potential is highest are presented first. The broad selection of suggestions allows participants to set goals for sustainable behavior changes quickly without restricting their sense of autonomy. To promote execution and automation, the behaviors are linked to triggers that are common in the users’ everyday lives. Self-monitoring and feedback for successful performance is expected to increase perceived competence. The use of the BitHabit application is assessed using the log data collected throughout the intervention. The technical functionality and feasibility of the application was tested during multiple test sessions among 10 representatives of the target group. The StopDia group members had access to the BitHabit application throughout its development, which helped in ensuring the technical functionality of the application with a variety of devices since the beginning of the development.

##### Face-to-face group intervention

The face-to-face group intervention includes six group coaching meetings (Table 3) organized in local health care centers. The groups are facilitated by nurses and other health-care professionals who have received a 2-day training program following the principles of strength-based behavioral coaching. This approach has been designed and tested in the GOAL lifestyle intervention [12, 34] and further developed in several other studies in Finland and other countries [35].

**Table 3:** Contents of the StopDia face-to-face group meetings

Meeting topics	Key points
1. Orientation to the StopDia group coaching	Get familiar with other participants and the program Information on type 2 diabetes and lifestyle factors in its prevention
2. Rhythm of daily life	Observation of the daily rhythms of eating habits, physical activity, sedentary behavior, stress, sleeping, and rest Tools for management of daily life Goal setting and planning: Actionable behavioral goals to improve rhythm of daily life
3. Let’s eat well and healthy	Self-monitoring and reflection of dietary habits Principles of a healthy diet Goal setting and planning: Actionable behavioral goals for diet
4. Enjoying physical activity	Self-monitoring and reflection of physical activity Principles of sufficient physical activity Goal setting and planning: Actionable behavioral goals for physical activity
5. Automating activity to everyday life	How can I nudge myself to healthy lifestyle? Goal setting and planning: Actionable behavioral goals for re-designing home environment to support healthy choices
6. Succeeding in lifestyle management, also after the StopDia study	Self-evaluating program outcomes Learning and insights for future Planning for maintenance of behavior changes

Group meetings 1–5 are planned to be fortnightly and group meeting 6 approximately one month after meeting 5

All nurses receive the StopDia Instructors Manual where the contents of each group meeting are described with participatory activities and lead questions. The participants receive the StopDia Participant Workbook. The duration of the group coaching program is 3–4 months, and there are 6–15 participants in each group. Each meeting contains 90 min of organized activity and 30 min of optional activity. All meetings follow a similar enabling and functional structure supporting participant autonomy and participatory action that includes 1) warm-up to the meeting topic; 2) identification of strengths of the participants, including former knowledge, skills, and healthy lifestyle habits related to the topic; 3) learning something new and getting ideas for lifestyle change; 4) formulation of behavioral goals and action planning; 5) identification of sources of support and help needed to carry out the plan; and 6) closing the meeting with commitment to action. Between the face-to-face meetings, lifestyle changes are supported by homework materials and tools, available in the StopDia Participant Workbook. The nurses report the realization of the group coaching by filling out a detailed questionnaire after every group meeting.

##### Control group

The control group receives a digital information package on lifestyle risk factors for type 2 diabetes and dietary and physical activity recommendations to decrease the risk of type 2 diabetes. They are also informed that they will have the opportunity to use the BitHabit application after the one-year follow-up.

#### Primary and secondary outcomes

The primary and secondary outcomes of the randomized controlled trial are changes in the corresponding variables during the 1-year follow-up listed in Table 4. Each of them are described in more detail in the following paragraphs.

**Table 4:** The primary and secondary outcomes of the randomized controlled trial

Primary outcomes	Secondary outcomes (in more detail below and in ClinicalTials.gov. Identifier: NCT03156478)
Body weight	Waist circumference
Fasting plasma glucose	Serum insulin and plasma lipids
Two-hour plasma glucose from OGTT	Systolic and diastolic blood pressure
Dietary score	Sedentary behavior
Total physical activity	Psychosocial factors and mental wellbeing Use of health and social care and associated costs

#### Assessments based on StopDia digital questionnaire at baseline and one-year follow-up

##### Sociodemographic factors

Date of birth, gender, marital status, education, employment status, the number of household members, household income, economic problems during the last 12 months, and ethnic origin are asked.

##### History of diseases and use of medications and health care services

Perceived health, the perceived risk of developing type 2 diabetes, the history of 32 non-communicable diseases diagnosed by a physician, the lifetime history of weight loss, perceived memory, adopting new knowledge, learning new things, the family history of type 1 and 2 diabetes, coronary heart disease, ischemic stroke, hemorrhagic stroke, hypertension, obesity, and dementia diagnosed by a physician, and diseases reimbursed by the social security system are asked. The use of health care services and prescribed medications during the last 12 months are also inquired.

##### Food, drink, and alcohol consumption and eating behavior

Food, drink, and alcohol consumption is assessed by 18 items that are slightly modified from a previously validated Finnish food intake questionnaire [36]. A composite diet quality score is created to reflect the goals of the intervention. The features of eating behavior are assessed using items from the ecSatter Eating Competence Inventory 2.0 (ecSI 2.0) [37] and the Three Factor Eating Questionnaire (TFEQ-R18, emotional eating factor) [38].

##### Physical activity and sedentary behavior

Conditioning physical activity, everyday physical activity, physical activity to and from work, physical activity and sitting at work, and sitting and other sedentary behavior during leisure time are assessed using questions modified from those of the Finrisk study [39, 40] and the Kuopio Ischaemic Heart Disease Risk Factor study [41] in Finland and the International Physical Activity Questionnaire [42].

##### Sleep behavior, smoking, and alcohol consumption

Sleep behavior, including duration, quality, timing, and regularity, is assessed by questions modified from the Basic Nordic Sleep Questionnaire [43]. Smoking habits are assessed using questions modified from the Finrisk 2012 survey [44]. Alcohol consumption is assessed by items modified from a previously validated Finnish food intake questionnaire [36].

##### Psychosocial factors

Motivation to eat healthy and do physical activity is assessed by the four best items of autonomous motivation and the four best items of controlled motivation from the Treatment Self-regulation Questionnaire [45]. We added two items of intrinsic motivation. Perceived competence is assessed by the Nutrition Self-Efficacy Questionnaire and the Physical Activity Self-Efficacy Questionnaire [46]. Self-regulation is assessed by the Brief Self-Control Scale [47], action and coping planning by items modified from the Action and Coping Planning Scales (only at one-year follow-up) [48], behavioral automaticity by the automaticity subscale from the Self-Report Behavioural Automaticity Index (only at one-year follow-up) [49], social support for physical activity and diet with a modified version of the Brief Social Support Scale (only at one-year follow-up) [50], and time orientation by four items modified from the 22-item Zimbardo Time Perspective Inventory [51]. Quality of life is assessed by the World Health Organization Quality of Life scale [52] and work ability and activity by the Work Productivity and Activity Impairment Questionnaire [53]. Mental wellbeing is assessed by the Warwick-Edinburgh Mental Well-being Scale [54], satisfaction with life by the Cantril’s Self-Anchoring Ladder [55], perceived stress by the Perceived Stress Scale [56], and resilience by the 14-item Resilience Scale [57]. Loneliness is assessed by the 12-item version of the UCLA Loneliness Scale [58, 59]. Questions used to assess cognition, including perceived memory and learning capability, are from Finrisk 2012 survey [44]. The dementia risk score is computed based on a previously created risk score [60].

#### Measurement of anthropometrics and blood pressure

Height, weight, waist circumference, and blood pressure are measured by trained nurses in local health care centers. Body weight is measured in light indoor clothing by digital scales to accuracy of 0.1 kg. Body height is measured in a Frankfurt position and without shoes by scaled height meters to accuracy of 1 cm. Body mass index (BMI) is calculated by dividing body weight in kilograms by the square of body height in meters. Waist circumference is measured in a standing position on bare skin at the end of normal exhalation and at the mid-distance between the bottom of the rib cage and the top of the iliac crest to accuracy of 1 cm. Resting blood pressure is measured after a 5-min rest two times with 2 min intervals from the right arm in a sitting position with standard automatic sphygmomanometers to accuracy of 1 mmHg. The means of the two measurements are used as systolic and diastolic blood pressure.

#### Laboratory measurements

Blood samples for measuring glucose and insulin metabolism (fasting, 30-min, and 2-h glucose and insulin from 2-h OGTT), lipid metabolism (fasting plasma total, LDL, and HDL cholesterol and triglycerides), liver function (serum aspartate aminotransferase and alanine aminotransferase), biliary function (serum alkaline phosphatase), kidney function (serum creatinine), and thyroid function (serum thyreotropin) are analysed in the standardized quality-controlled clinical laboratories of the three provinces or in the laboratory of the University of Eastern Finland. The disposition index_30_ (DI_30_) is used as a surrogate measure of early-phase insulin secretion, and the Matsuda index of insulin sensitivity (Matsuda ISI) is used as a surrogate measure of peripheral insulin sensitivity, as previously validated against the 2-h OGTT [61–63]. Blood samples for genetic analyses and metabolite profiling will be analysed in the research laboratory of the University of Eastern Finland.

#### Assessment of the use of health care services and medications and health economic evaluation

The use of health care services in outpatient clinics of hospitals, public health care centers, occupational health care centers, and private health care providers as well as at home and the use of medications of the participants during the last 12 months are asked in a questionnaire. The use of health care services and medications and associated costs are also received from Care Register for Health Care (outpatient and inpatient visits in hospitals) and Register of Primary Health Care Visits (outpatient and inpatient visits in public health care centers) of National Institute for Health and Welfare of Finland and Finnish Prescription Register (reimbursed medicines) and other registers of Social Insurance Institution of Finland (other costs related to health care) using national personal identity codes. The long-term cost-effectiveness of the interventions is assessed using health econonomic modelling, which makes it possible to simulate the expected health outcomes, costs, and cost-effectiveness of the interventions in the long term based on the observed short-term changes in risk factors for type 2 diabetes. The results of the health economic modelling will be confirmed by the register-based cost-effectiveness analyses in the future.

#### Power and sample size calculation

The estimation of the number of individuals needed in the randomized controlled trial is based on power calculations relying on the net difference of 0.6 mmol/l in the change of 2-h plasma glucose during the first 12 months of follow-up between the intensive lifestyle intervention group and the control group in the Finnish Diabetes Prevention Study [1]. Due to the real-life community-based approach in the StopDia study, we realistically anticipate achieving about 0.3 mmol/l net difference in the change of 2-h plasma glucose during the 12-month follow-up between the intervention group and the control group. We estimate that a minimum number of participants required to detect an effect size of at least 0.2 mmol/l in the change of 2-h plasma glucose concentration with a power of 80% (*p* = 0.05, allowing for a 10% loss to follow-up or missing data) is 1000 participants in each group.

#### Statistical analyses

The statistical analyses will be performed following the CONSORT 2010 statement guidelines for randomized controlled trials. The effects of the intervention on the primary and secondary outcomes during follow-up are analysed using the intention-to-treat principle. Linear mixed effects models adjusted for relevant confounding factors are used to analyse the effects of the interventions on the outcomes. Appropriate correction methods are used to reduce the likelihood of false positive findings in the statistical analyses. Differences in variables between the groups and associations and interactions between variables with *p*-values of < 0.05 are considered statistically significant. Most of the statistical analyses are performed using the IBM SPSS Statistics® software (IBM Corp., Armonk, NY, USA), but the SAS and R software may also be used.

### Protocol for evaluating adoption, implementation, and maintenance

Adoption and implementation of the interventions will be evaluated with project logs, group facilitator logs, questionnaires, and interviews (setting level) as well as key informant interviews (societal level). Participation, experiences, and acceptability [64] dealing with the digital intervention and the face-to-face group intervention as well as experiences dealing with the StopDia study are assessed at the one-year follow-up questionnaire. We developed a seven-item questionnaire of acceptability based on the Theoretical Framework of Acceptability [64]. User data from the BitHabit application is used for measuring adherence, habit selection, and habit execution.

### Data management

Collected data will be stored in secure file servers at the University of Eastern Finland. The file servers are located in a locked and monitored server room. All data can be accessed only by users authorized by the principal investigator. Personal identification numbers will be only used to combine data from different sources, and access to these data will be restricted to within the University of Eastern Finland firewall. Coded data with identifiable information removed will be used for statistical analyses. At the end of the study, the data will be stored by the University of Eastern Finland or CSC - IT Center for Science.

### Ethical issues

The StopDia study was approved by Research Ethics Committee of Hospital District of Northern Savo (statement number: 467/2016). Written informed consent to participate in the study and the use of data from national health care registers is obtained from all participants. The study will be conducted according to the Responsible Conduct of Research by the Finnish Advisory Board on Research Integrity and the Declaration of Helsinki. The participants were permitted to have their usual care during the study. After the randomized controlled trial, the control group will be provided a possibility to start using the BitHabit application.

### Study management

The principal investigator (PI) Jussi Pihlajamäki leads the Study Management Team that includes the leaders of all Work Packages (Jussi Pihlajamäki, WP 1: Coordination and general management; Pilvikki Absetz, WP2: Behavior change at individual and environmental level; Jaana Lindström, WP3: Identification of individuals at increased risk of type 2 diabetes; Miikka Ermes/Marja Harjumaa, WP4: Digital solutions to support individuals in healthy lifestyle; Co-PI Timo Lakka, WP5: Individual level intervention; Kaisa Poutanen, WP6: Environmental intervention to support healthy lifestyle; Johanna Kohl/Janne Martikainen, WP7: Socio-economic barriers and facilitators to support decision making) and the study coordinator Tanja Tilles-Tirkkonen. The Study Management Team is responsible for overall study management as well as the progress and approval of scientific and financial reports. The Scientific Advisory Board includes international scientific experts, Professor Peter Schwarz, Professor Edith Feskens and Professor Theresa Marteau. The Scientific Advisory Board reviews strategic decisions, follows study implementation, and critically evaluates study results.

### Dissemination policy

We will publish study results in high-quality peer-reviewed international scientific journals and will prefer open-access journals. The publications will also be made openly available through parallel publishing in the publication archive of University of Eastern Finland. We will publish the most important findings in international scientific meetings as well as national scientific meetings and journals. Our articles in peer-reviewed international journals and our doctoral theses will be published on the website of University of Eastern Finland according to its publication policy. We will deliver almost all of our findings to the media through press releases, our webpages, and Twitter to disseminate them to health professionals, society decision makers, and the public.

## Discussion

The StopDia study is based on the convincing scientific evidence that type 2 diabetes and its comorbidities can be prevented by a healthy lifestyle. The reason for the need for additional research is that the attempts to translate scientific evidence into action in real-world health care have not led to permanent and cost-effective models for the prevention of type 2 diabetes. The StopDia study will complement previous research by 1) exploring how to improve reaching individuals at risk, 2) studying how to increase participation in and adherence to lifestyle intervention, 3) creating personalized intervention models, 4) evaluating the efficacy and cost-effectiveness of actions to improve lifestyle and prevent type 2 diabetes, and 5) promoting the adoption, implementation, and maintenance of the preventive actions that are fully intergrated in the Finnish routine health care. This will enable us to leverage our societal objective of developing a participatory, sustainable, and scalable prevention model for preventing type 2 diabetes that engages citizens, societal actors, and other stakeholders.

### Identification of individuals at increased risk of type 2 diabetes

One of the challenges in the prevention of type 2 diabetes has been how to reach individuals at increased risk of type 2 diabetes. Although knowledge on the risk factors and prevention of type 2 diabetes have increased in the society, there still are many people who do not recognize being at increased risk. To reach these individuals, we have developed a simple and non-invasive tool to reach these individuals. In the StopDia study, the FINDRISC is available in a digitalized format and to be completed at any time. However, providing the opportunity to screening individuals at increased risk is not enough. We need efficient communication strategies to increase the reach and uptake of risk identification, both within and outside health care services. In the StopDia study, we provide scientific evidence for the effects of the different recruitment approaches and emphasize reaching individuals from different population groups, including those with a lower socioeconomic position.

### Increasing motivation to participate in and adherence to lifestyle interventions

Ultimately, success to prevent type 2 diabetes at individual and societal level depends on the ability of the program to increase the adherence of participants to a healthy lifestyle. To achieve this, lifestyle interventions have largely focused on the motivation and capability of the participants. Although supporting autonomous motivation and self-regulation skills, such as goal setting and planning, have been proven to be effective in the short-term, the sustainability of lifestyle changes remains a challenge [65]. The theories of habit formation and automatisation offer potential solutions for sustainability. Based on habit theories, simple actions are easier to conduct and adopt if they are linked to a stable context and are repeated until they become automatised [28, 29]. Habit theories are suitable for digital interventions where simplicity and effortlessness are key factors. Habit theories were chosen for the StopDia BitHabit intervention tool. The advantages of the digital tools compared to traditional options are 1) the possibility to objectively measure adherence to the interventions, 2) the low cost of the interventions, and 3) that the interventions are not tied to time or place and are therefore flexible. On the other hand, there are still individuals who have no access to digital services or for whom such tools are not suitable. Especially for these individuals, face-to-face group lifestyle coaching might still be the most suitable and cost-effective choice for the prevention of type 2 diabetes.

### Creating personalized intervention models

Personalised approaches are needed to increase the commitment of individuals and the effectiveness of the interventions. The StopDia study enables us to explore interventions that could be suitable to different individuals and population groups and create a model to predict responses to the interventions. For the future, it will be of great importance that the model with its interventions can reach individuals from different cultural, ethnic, and socioeconomic backgrounds. Although the randomized controlled trial in the StopDia study could not include diverse populations with different needs, we are simultaneously adapting and piloting the model in two vulnerable population groups, immigrants and long-term unemployed people.

### Integrating the StopDia model to the society

To ensure the implementation of the StopDia model into real life we have closely collaborated with over 20 social and health care partners from the planning to realization of the study. Importantly, we are able to evaluate the effectiveness and cost-effectiveness of the actions to prevent type 2 diabetes as part of the routine health care in the real world environment. Combined with the co-creation of the actions, we are promoting adoption, implementation, and maintenance of the preventive actions as part of the routine health care.

Due to the limited economic resources, it is very important that the model created is effective and financially sustainable. Therefore, the direct link of the StopDia data with routine clinical and biochemical data, biobanks, and registries measuring the outcomes of the project is essential. Furthermore, the digital screening of individuals at increased risk, the collection of data on risk factors for type 2 diabetes, and intervention tools created in the StopDia study gather information that is not normally collected in the health and social care and could therefore be very useful in preventing type 2 diabetes in the future.

Sustainable and scalable models are needed to reach and identify individuals at increased risk of type 2 diabetes and to deliver personalized and effective lifestyle interventions. With the StopDia study we aim to answer these challenges in a scientific project that is fully integrated in the routine health care and thus allows the real-life assessment of cost-effectiveness of the intervention. Even though the StopDia study focuses on the prevention of type 2 diabetes, the same lifestyle goals and tools are effective in the prevention of other non-communicable diseases, such as cardiovascular diseases, dementia, osteoarthritis, and cancer. Therefore, the potential of the StopDia approach extends beyond the prevention of type 2 diabetes.

## Abbreviations

BMI: Body mass index
cm: Centimeter
DI_30_: Disposition index_30_
ecSI 2.0: The ecSatter Eating Competence Inventory 2.0
FINDRISC: The Finnish Diabetes Risk Score
HDL: High-density lipoprotein
kg: Kilograms
LDl: Low-density lipoprotein
Matsuda ISI: Matsuda index of insulin sensitivity
mmHg: Millimeter of mercury
mmol/l: Millimoles per litre
OGTT: Oral glucose tolerance test
PI: Principal investigator
RCT: Randomized controlled trial
RE-AIM: The Reach, Effectiveness, Adoption, Implementation, and Maintenance framework
StopDia: Stop Diabetes study
T2D: Type 2 diabetes
TFEQ-R18: The Three Factor Eating Questionnaire
WP: Work package
